# Irish Nursing Affairs

**Published:** 1918-06-08

**Authors:** 


*To the Editor of* THE HOSPITAL.

### Irish Nursing Affairs.

### THE FACTION SOWERS EXPOSED AND REBUKED.

*To the Editor of* THE HOSPITAL.

SIR,—Surpassing the limits of good taste, a contemporary journal has thought fit to associate the name of Lady Arnott with Irish nursing faction. It is suggested that in connection with the proposed Irish Tribute Fund for Irish Nurses Lady Arnott deliberately omitted to invite the President of the Irish Nurses' Association to attend the initial meeting. Nobody acquainted with Lady Arnott and her twenty years of patriotic labour on behalf of disabled sailors and soldiers, not to mention the Red Cross, will give the slightest credence to any such suggestion. She is one of Ireland's broadest-minded and most patriotic workers. From motives of pure patriotism she has taken up the cause of Irish nurses, and even to hint that she is associated with pettiness or faction is an impertinence that can only be attributed to ignorance.

It may interest readers of THE HOSPITAL to know that the issuing of invitations to attend the meeting referred to was absolutely and altogether in the hands of the organiser, Mr. Henry McLaughlin. Mr. McLaughlin is a man of the world in the broad sense, and from my personal knowledge I can testify that he knows nothing, and prefers to know nothing, of the domestic and somewhat squalid quarrels for supremacy fomented by some of the so-called leaders of the rank and file. He is out to benefit the Irish nursing profession, and he has undertaken to collect £10,000, part of a great national fund, with the proviso that the £10,000 referred to shall be earmarked for Ireland. It is a big undertaking, and I hope his efforts will be crowned with success. Now at the initial meeting Mr. McLaughlin unfolded his scheme, and he issued his invitations to anybody he thought of who in his opinion might be able to help the movement forward. Public men and women in high social, professional, and commercial circles were invited, and, so far as the nursing profession is concerned, he included the matrons of all the Dublin hospitals. I doubt if Mr. McLaughlin ever heard of the Irish Nurses' Association, but in any case his invitation included its most active and leading members, and from these letters of apology for non-attendance were read. One of the most prominent has been invited to serve on the Executive Committee. It so happens that the President for the current year has for a long time retired from active service. She is a Scottish lady, trained in England, and was some years ago a Dublin hospital matron. Had she been in active service when the invitations were issued she would have been invited with her colleagues.

It is earnestly to be hoped that we are not to have faction fighting introduced into this Irish Tribute to Irish Nurses. It is surely a case of *noblesse oblige*. The only people who can help the movement are those who can either contribute money or influence money contributions. No doubt the patriotic aim of some people who can do neither is to administer the fund when other people collect it, and, if they see no prospect of achieving their ambition as administrators, to do all in their power to paralyse the efforts of the workers. I am quite sure, and this I say with deliberation, that anybody associated with or interested in the nursing profession in Ireland who can even in a small way help to make it a success will be warmly welcomed by Mr. McLaughlin. He is out to accomplish his undertaking—to benefit the trained nurses of Ireland—nothing more and nothing less.

**The corollary is obvious. Individuals who choose to stand aloof because of some purely imaginary slight are allowing their personal considerations of dignity and importance to stand in the way of a benefit fund for the rank and file. This is the root cause of faction in the profession, and the nurses of Ireland will do well to make careful note, during the campaign which is about to be conducted on their behalf and for their personal benefit, if and from what quarter opposition comes, and by what motives such opposition is inspired. All the money collected in Ireland will be spent in Ireland. Yet the prime mover in the business is an Englishman.**

IERNE.

